# Recommendations to Enhance Engagement in Perinatal Research With People Who Inject Drugs

**DOI:** 10.1111/dar.70283

**Published:** 2026-10-21

**Authors:** Alison D. Marshall, Grace K. Burton‐McKeich, Natalie Taylor, Kristen Overton, Antonia W. Shand, Alys Havard, Danielle M. Russell

**Affiliations:** ^1^ The Kirby Institute University of New South Wales Sydney Australia; ^2^ Centre for Social Research in Health University of New South Wales Sydney Australia; ^3^ Implementation to Impact (i2i), School of Population Health University of New South Wales Sydney Australia; ^4^ School of Clinical Medicine, Faculty of Medicine and Health University of New South Wales Sydney Australia; ^5^ Department of Infectious Diseases Prince of Wales Hospital Sydney Australia; ^6^ Children's Hospital at Westmead Clinical School, Faculty of Medicine and Health The University of Sydney Sydney Australia; ^7^ Department of Maternal Fetal Medicine Royal Hospital for Women Sydney Australia; ^8^ National Drug and Alcohol Research Centre University of New South Wales Sydney Australia

**Keywords:** community‐engaged research, health equity, hepatitis C, people who inject drugs, perinatal research, pregnancy, women

## Abstract

Women of reproductive age are significantly less likely than men to initiate hepatitis C virus (HCV) treatment, with the lowest uptake among women with children. Women who inject drugs who have children are a highly marginalised and stigmatised population who experience inequitable access to healthcare and poorer health outcomes. In the BUMP‐C qualitative study—*Bridging Unified Management of Pregnancy in hepatitis C care*—we sought to investigate current HCV care pathways and perceptions of HCV management during pregnancy among women who inject drugs. During this study, several lessons emerged from both the challenges and successes in engaging with this population. The aim of this commentary is to outline key recommendations for optimally engaging women who inject drugs in perinatal research. These recommendations include building trust with research participants, promoting strategies for research flexibility and seeking and valuing community and lived experience. The voices of women who inject drugs, often neglected in this space, must be prioritised to improve health outcomes for them and their children.

## 1 Stigma, Discrimination and Inequities Experienced by Women Who Inject Drugs

People who inject drugs are a marginalised and stigmatised population. Their life experiences are often shaped by intersecting harms—physical and sexual violence, homelessness/unstable housing and incarceration—that are embedded within various structural drivers such as gender inequity, poverty and criminalisation [1, 2, 3, 4]. Accordingly, they frequently experience poorer health outcomes—for example, reduced quality of life, higher risk of infections (e.g., human immunodeficiency virus [HIV], hepatitis C virus [HCV], skin and soft tissue infections) and premature death [2, 5, 6]. Services and support, if available, are often not structured to accommodate their needs, making access logistically and financially challenging (e.g., limited operating hours, geographically dispersed services) [7]. They frequently experience stigma in healthcare settings due to provider attitudes and inflexible institutional practices (e.g., restrictive takeaway opioid agonist treatment policies), which reduces trust and creates a mismatch between the low‐threshold harm reduction services that people who inject drugs need and the services offered [8, 9, 10, 11]. As a result, many will avoid or delay seeking care in primary and acute settings (until their condition is more advanced) and discharge themselves early from hospital [12, 13, 14, 15, 16, 17, 18, 19, 20].

Intersectional factors affect equitable access to healthcare and highlight groups who require targeted support services [21]. People who inject drugs *and* are Indigenous, homeless and/or are women or non‐binary can experience greater health disparities—for example, less likely to receive HCV treatment—than those without these characteristics [22, 23, 24, 25]. Criminalisation of drug use makes women who inject drugs especially susceptible to systemic stigma and discrimination [26]. In parallel with the broader literature, our research has demonstrated that women who inject drugs often report gender‐based ‘double‐stigma’ for being a woman who uses illicit drugs and does not conform to traditional gender norms of womanhood [22, 27, 28, 29, 30]. Women who engage in the perceived masculine realm of drug use are viewed by society (and sometimes themselves) as amoral, unclean, unfeminine, unattractive and promiscuous [22, 29, 31, 32]. Women who inject drugs and have children report heightened social stigma, compared to men/fathers, for not modelling ‘good mother’ behaviour, despite repeatedly stating an interest in keeping their children safe and seeking access to substance use treatment support (e.g., residential treatment) in the face of immense system‐level barriers (e.g., extensive wait lists, no childcare onsite) [22, 26, 27, 32, 33, 34]. This cohort continually encounters a double‐bind in service settings: public health guidance encourages disclosure of drug use, yet heightened surveillance, mandatory reporting and the risk of child removal have been identified as system level barriers to disclosing their drug use, even when they may want help [26, 33, 34]. Among this population, who have very likely experienced some form of child and/or current trauma (e.g., domestic violence), such systems make it exceedingly challenging to engage in care and build client‐provider trust [3, 29, 35].

## 2 The BUMP‐C Study

As researchers and clinicians who study or provide services to women who inject drugs [27, 36, 37], we had a firm understanding of such factors when implementing the BUMP‐C study—*Bridging Unified Management of Pregnancy in hepatitis C care*. For this pilot qualitative study, we interviewed participants who were: 18 years or older; women, trans men, trans masculine people, non‐binary or gender diverse; gave birth since 2016 and/or planning a pregnancy in the next 2 years; have been tested or treated for HCV; have experience with injection drug use (current or past); willing to have their interview audio recorded; able to speak and understand English well enough to participate in an interview. Manuscript author Russell identifies as a woman and has living experience of injection drug use and opioid agonist treatment. In some ways, Russell would be defined as a ‘peer’ to the research participants in the BUMP‐C study [38], though she does not have child‐rearing experience. However, based on peer research in the harm reduction literature, it was anticipated that the interviewer–participant would have enough shared experience to develop relational trust and ‘safe care spaces’, creating a sense of belonging for participants in the research process (discussed in Recommendation 1) [39, 40, 41, 42].

The BUMP‐C team was intentionally constructed to include living experience as well as diverse but complementary expertise to investigate the clinical care gaps experienced among women who inject drugs in the perinatal field. This included clinical specialities (obstetrics, infectious diseases), HCV and addiction research, implementation science and living experience, as well as different methodological expertise (e.g., quantitative, qualitative, mixed‐methods). For this pilot study, since HCV infection served as its primary focus (due to funding and timeline constraints), we were unable to be as ambitious in research scope as we would have liked. Our approach towards this study had to remain narrowly focused on HCV‐related care, but we also recognised—and indeed, it was reflected in participant narratives—that wider system‐level challenges experienced by this cohort warrant further attention (e.g., lack of housing supports). This process collectively underscored to the BUMP‐C team the broader work that needed to be done (e.g., improved opioid agonist treatment access during pregnancy and postpartum) via a larger grant that would permit us to not only address these structural barriers more systematically but also permit each researcher the opportunity to investigate these challenges through several separate studies (e.g., an implementation study on testing a peer navigator program). Given this, the BUMP‐C team intends to apply for further funding to investigate these care gaps and address broader participant needs.

In this commentary, we will draw on lessons learned from the research challenges encountered during the BUMP‐C study. We will also reflect on the study's successes. For example, one of our primary research challenges was the lengthy period of participant recruitment: over a 12‐month data collection period, we interviewed 18 women and one non‐binary person. Based on our prior research experience, this prolonged process was unexpected and prompted us to reflect on how we could have strengthened the BUMP‐C study overall. It was during this reflection that we also noticed unanticipated positive outcomes. Participant recruitment began to occur via word‐of‐mouth (without our direct involvement), and the data obtained were rich and illuminating. Indeed, we began to observe how specific challenges and benefits could be categorised under general themes that, in turn, became recommendations or guiding principles for how we would like to conduct future research. The broader literature has investigated barriers and facilitators to recruitment and retention of pregnant women with substance use challenges [43, 44, 45], often with a specific focus (e.g., longitudinal research, data confidentiality) [44, 46]. Building on these more targeted considerations, this commentary presents broader recommendations for conducting research with women who inject drugs, particularly in the perinatal period, with the aim of supporting more ethical, inclusive and responsive research practices. While we did not strategically apply a trauma‐informed lens when conducting the BUMP‐C study, we agree with more recent calls to apply such a framework (e.g., providing safety and trustworthiness, offering choice) when working with marginalised population groups who have experienced trauma [47]. Where appropriate, we will make linkages to trauma‐informed principles in the recommendations below. By sharing these lessons, we hope to enhance the engagement of women who inject drugs in future research and to amplify their voice in the evidence base—which has been neglected for far too long [31]—with the ultimate goal of improving services and health outcomes.

## 3 Recommendation 1: Build Trust With (Potential) Participants

Similar to prior research [43], we recognised the importance of building trust with participants and hence, implemented several strategies to signal trust. As noted, Russell is a peer to participants although she does not have child‐rearing experience. To help address this ‘gap’, the BUMP‐C investigator team developed a Community Expert Panel and collaborated closely with a national drug user organisation—both of which involved women who inject drugs with experience of perinatal care (discussed in Recommendation 3).

Given the sensitive subject matter of the BUMP‐C study (perinatal care, history of injection drug use and HCV infection) and peer worker and harm reduction literature [39, 40, 41, 42], we anticipated that this participant cohort would feel more at ease and have greater trust in a peer interviewer. Russell therefore scheduled and conducted all interviews. This approach helped to establish trust, continuity and rapport with participants. We allowed for the possibility that some participants may have preferred a non‐peer interviewer, yet no participant opted to be interviewed by a different member of the research team. Throughout participant recruitment, we highlighted Russell's role as interviewer through various strategies, for example, word‐of‐mouth, listing her name on the study flyer and having her schedule the interviews. We also underscored this to clinic staff when asking if they would advertise the study flyer at their services.

In addition to creating a trustworthy space with a peer interviewer, we believe (although this is not the primary argument) that the data retrieved were likely richer and of greater depth than if a non‐peer conducted the interviews. A peer interviewer also likely led to participants feeling more comfortable taking part in research and, in turn, may increase their willingness to take part in future research—an important point given that women who inject drugs remain underrepresented in the literature [31], especially in the perinatal field.

Still, even with a peer interviewer, we did not anticipate the level of trepidation participants would exhibit prior to enrolling in the BUMP‐C study. Most participants spoke to Russell on multiple occasions about the study before enrolment. For us researchers, this experience had not occurred before. Russell had been simultaneously conducting qualitative interviews with peer workers from harm reduction and HCV‐related services (*n* = 27 interviews) in a much shorter timeframe and without the same degree of pre‐interview workup, and it became clearer that participant recruitment for the BUMP‐C study would take longer than prior studies. Compared to participants in the peer‐worker study, we think that participants in the BUMP‐C study were much more likely to be distrustful of services, to ‘suss out’ Russell's intentions and use more caution given that the study concerned perinatal care specifically (some participants may have had experience with child protection services and could have been concerned as to whether Russell worked for such services, whether she would report interview discussions to protective services or whether their participation in the study would affect their clinical care).

It also became increasingly evident that the majority of enrolled BUMP‐C participants had never participated in a research study before. Participants were especially unclear about what the research entailed and how findings would be presented to protect confidentiality (e.g., concerns that the transcribed research interviews would be published in their entirety and publicly accessible). Consistent with prior research [46], Russell took the time to explain the BUMP‐C study and simplify the details of the study protocol to build a ‘sense of trust’—in effect, acting as a ‘research translator’ [43]. This process underscored to us that timelines and budgets for research with this population (and other marginalised groups) may need to accommodate a lengthier recruitment process, giving researchers the time needed to apply a trauma‐informed lens and create trustworthy environments for research participation [47].

Another strategy we implemented to instil trust was to offer participants cash via direct deposit rather than vouchers, which required minimal personal information (e.g., payment identifier). We learned that providing the reimbursement to participants post‐interview but while they were still on the telephone or online was preferred, as participants could see the cash transfer instantly. This was appreciated by participants, as many were struggling financially and we think it reduced their anxiety as to whether they would be reimbursed (again, participants were new to research and may have been unsure if we would ‘keep our word’). Researchers and the ethics committee should consider the use of cash or direct deposit to reimburse participants. We believe that participation would have been lower had vouchers been offered instead. In agreement with the literature [43, 48, 49, 50], we believe that cash reimbursement underscored our respect for participant dignity and autonomy, signalled trust and offered practical support in a way that vouchers do not.

Given the duality of Russell's role (academic and peer), we believe that this contributed to more immediate participant–interviewer connection (which aligns with trauma‐informed frameworks) [47]. Still, similar to peer literature in harm reduction, this setup could increase the likelihood of compassion fatigue and feelings of powerlessness in interviewers being unable to assist participants beyond this study (e.g., housing support) [42, 51]. In addition, as women and gender diverse people who inject drugs comprise a heterogeneous group, there may be circumstances in which participants and interviewers disagree (e.g., opioid agonist treatment) and express statements perceived as stigmatising [31, 32, 52]. As more research includes the expertise of people with lived and living experience, research teams and ethics committees should consider how to best support the interviewers in the same way that a ‘do no harm’ principle applies to participants [53]. We need guiding documents in which support can be better extended to those in the research space with shared experiences to the participants, particularly among those who may share stigmatised identities, including those in fields beyond harm reduction (e.g., lung cancer, weight loss) [42, 54, 55, 56].

Finally, while we sensed that many potential participants seemed distrustful of people beyond their immediate social circle, it became clearer during the interviews that many participants lacked strong familial or friend networks. Participants in the BUMP‐C study may have been especially marginalised, requiring additional assurance before trusting us with their experiences or histories. Following the underlying principles of trauma‐informed research [47], we needed to implement greater elements of *research flexibility*, that is, offer participants greater choice in how we collected our research data while still upholding ethical standards.

## 4 Recommendation 2: Promote and Develop Strategies for Research Flexibility

In a post‐COVID‐19 world, we believed—along with other researchers in the field—that conducting our qualitative interviews via telephone or online would enhance equitable access to research [57]. This approach, utilised with our other qualitative studies in the drug and alcohol field [58, 59, 60, 61], has increasingly become standard research practice. Yet partway through data collection, it became evident that some participants would have preferred an in‐person interview. We should have offered this option to participants living in Sydney (where we are based) and more generally, as opportunities arose.

For instance, when Russell attended a national viral hepatitis conference in Melbourne, there were potential participants at the conference (and in the surrounding area) who stated a preference for an in‐person interview. The BUMP‐C study only had ethics approval for online interviews, and while we could have applied for an amendment to our ethics approval, we were concerned that this might delay study recruitment further. In such circumstances, Russell needed to schedule a telephone or online interview even when the potential participant was available in person. It is possible that offering the option of an in‐person interview would have given participants the additional assurance that Russell could be trusted. Providing greater options for research participation would have also likely reduced the data collection period. In‐person interviews are not necessarily more inherently ‘burdensome’ for the participant; for example, if the interviewer can reimburse transport costs and parking, provide a safe and private space for discussions and even offer coffee and light refreshments pre‐ or post‐interview [43, 46].

Offering only telephone or online interviews also limited opportunistic recruitment. As part of the National Hepatitis C Testing Program, there are campaign days that involve staff entering a service to help facilitate HCV testing and treatment initiation among people who inject drugs [62]. On a few occasions, Russell attended these campaign days to promote the BUMP‐C study. Some participants appeared willing to be interviewed on‐the‐spot but seemed more hesitant to telephone the study number to arrange an interview. The requirement to use a telephone or computer was also burdensome for participants facing economic and mental health challenges that affected their ability to maintain access to a mobile phone. For example, Russell needed to help coordinate telephone access for a participant. This points to potential barriers to research participation given that qualitative studies are increasingly utilising ‘everyday’ technologies (e.g., video platforms).

For future studies, we recommend offering both online and in‐person interviews wherever possible. While implementing several interview approaches may have its drawbacks (e.g., lack of interviewer observational capacities during online interviews; reduced geographical representation for in‐person interviews), we believe that providing as much flexibility in *how* research is conducted is the most *equitable* approach. This approach applied to recruitment, where participants were offered multiple ways to be in contact with Russell and learn about the study (e.g., email, text, telephone), so that participants could use their preferred method.

Other strategies that we implemented to promote and develop research flexibility included giving participants multiple options for *when* they participated in the interview. Interviews could be scheduled beyond standard business hours, at weekends, or on‐the‐spot (e.g., when someone telephoned to ask about the study). We also emphasised to participants that the video camera was optional for interviews (i.e., not part of inclusion criteria). Some researchers might argue that this would have hindered our ability to observe participant reactions to interview questions. Nonetheless, permitting as many options as possible—again, consistent with applying a trauma‐informed approach [47]—likely instilled interviewer–interviewee trust more rapidly, and in turn, elicited more in‐depth responses.

## 5 Recommendation 3: Seek and Value Community and Lived Experience

For the BUMP‐C study, we intentionally sought input from the community and people with lived and living experience. This point might seem obvious, but globally, researchers (and grant agencies) generally do not systematically call for such insights. Part of this may be due to uncertainty about how to engage meaningfully with community, but it is also likely due to narrow research priorities that undervalue non‐academic outputs, broad partnerships and research impact, especially when not required by funders. In addition—and as asserted by the activist and analytic lens of narcofeminism—the perspectives of women and gender diverse people who inject drugs have been historically undervalued in harm reduction, with such populations deemed too ‘weak’, ‘disordered’ and ‘irrational’ [30, 31]. Even though the BUMP‐C study was a pilot, we recognised from the outset that community input—that is, women and gender diverse people who inject drugs—was needed to keep the research study ‘on track’ conceptually, methodologically and analytically. A Community Expert Panel can provide guidance on participant recruitment, offer critical insight into participant narratives and identify community‐informed priorities for future interventions, including which current support and services are considered acceptable.

Thus, as part of the BUMP‐C study, an online Community Expert Panel was established with four women from across Australia with experience in injection drug use, HCV, pregnancy and motherhood. Panellists contributed as community representatives with living and lived experience, though some also held professional roles in harm reduction. They were identified through existing community networks and word‐of‐mouth recommendations. Although panellists did not take part in the BUMP‐C study, they were ‘peers’ to the enrolled participants, and we therefore made deliberate efforts to facilitate their engagement, applying the trust‐building and flexibility strategies used in the research space. Russell led the Panel, with four meetings held over 1 year. Having the Panel chaired by Russell created a trusted space for panellists to speak openly and confidently about their experiences in perinatal care—an opportunity novel to panellists—and helped inform specific aspects of the BUMP‐C study (discussed below). Russell also provided multiple reminders via text messages, email and telephone for upcoming sessions, recognising that panellists had preferred different modes of communication. One panellist preferred to keep her video camera off for the first session, which was accommodated without any issue. This flexibility in study implementation likely contributed to the Panel's success in developing an environment where women who inject drugs felt safe speaking about their past and current experiences.

Each Community Expert Panel meeting followed a different question guide developed by study investigators Marshall and Russell. Panellists were paid AU$100 per 60‐min session or AU$150 per 90‐min session (direct deposit). The Panel discussions informed the BUMP‐C study across three main domains: (i) participant recruitment; (ii) participant narratives; and (iii) future interventions. First, panellists identified potential services and strategies for participant recruitment and brought to light broader gaps in gender‐responsive care within harm reduction services. It was not always clear to panellists which services would be most appropriate for participant recruitment, given variable provision of gender‐specific care in harm reduction services—an area in need of further research. Second, panellists shared their experiences of perinatal care and provided important contextual information for interpreting participant data. Their attention to specific topics gave us a deeper understanding of the weight and salience of these topics for women who inject drugs (e.g., opioid agonist treatment during pregnancy), identifying areas of the participant narratives that would be important to focus on both during interviews (via prompts) and data analysis. Panellists' experiences, which often elucidated stigma and discrimination in service settings, also underscored to our research team why potential participants were initially distrustful of Russell prior to study enrolment. This re‐emphasised to us the importance of being patient and transparent prior to informed consent. Third, we asked panellists to speak to the types of perinatal interventions they envisage for women who inject drugs. Notably, while panellists could describe the services, they deemed *not* acceptable to their needs, they found it much more challenging to articulate services that were acceptable. It can be challenging to imagine hypothetical models of care, and for future Community Panels, we would be more specific about interventions or aspects of interventions to facilitate detailed discussion. Finally, ideally, we would have consulted the Panel prior to the ethics submission to provide feedback on the interview guide, but it took time to convene the Panel, and this did not occur. Given the immense benefit of community expertise, we recommend establishing the Panel sooner in the study process to allow for earlier contributions to the research study.

Our study team also reached out to collaborate with a national drug user organisation to seek their input. At the outset, we outlined expectations regarding roles and responsibilities for both organisations, and how we could work together most effectively. Marshall and Russell met monthly with this organisation to discuss multiple aspects of the study (e.g., participant flyer, interview guide, participant recruitment), and to keep the organisation updated on progress. In addition to creating opportunities to formally engage and facilitate study feedback, this approach also fostered collaborations on subsequent grants (e.g., the CARE study on reproductive health and people who inject drugs). Moving forward, we will continue to work with them to identify optimal ways to disseminate and translate findings to harm reduction services across Australia and globally, to help improve perinatal care for women and their children.

## 6 Recommendations

Taken together, the recommendations in this commentary provide guidance on conducting research with this population, including approaches that support meaningful participant engagement, strengthen recruitment and improve data quality. While there were elements of these recommendations that were consistent with a trauma‐informed lens, it is equally possible that a research framework is informed by and draws from other approaches (e.g., a relational ethics framework could provide researchers with a starting point when working with marginalised populations in health) [47, 63]. We also believe that the recommendations summarised in Figure 1 hold cross‐study and population appeal and are not necessarily specific to qualitative research, perinatal research, or to women who inject drugs. They could be considered when conducting research with marginalised populations more generally. While we recognise that researchers face system‐level constraints in conducting research that is co‐created with, or informed by, community (e.g., strict timelines for spending funds, which is not often conducive to partnership building), we need to keep advocating for ethical research with marginalised populations so that these recommendations become more common practice rather than aspirational.

**FIGURE 1:**
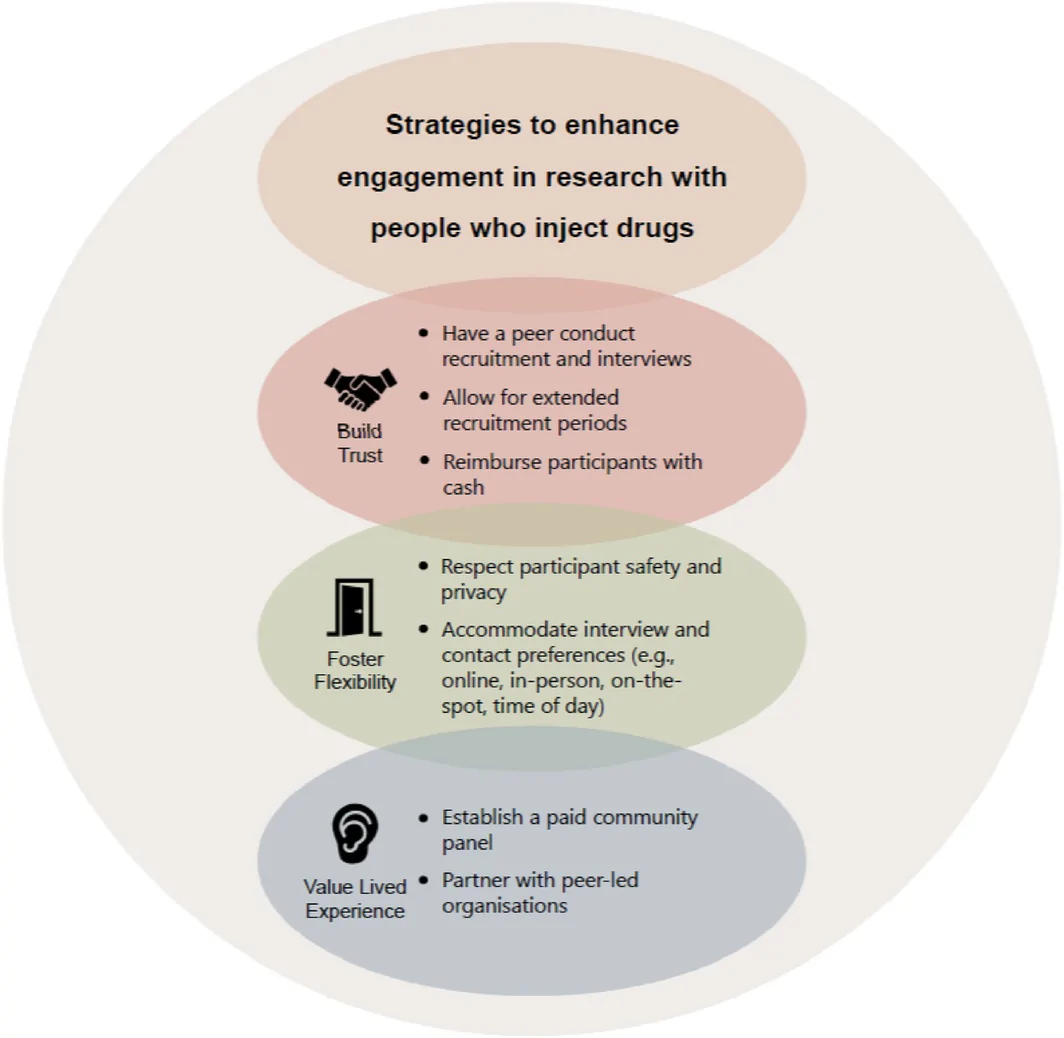
Recommendations to enhance engagement in research in people who inject drugs.

## 7 Conclusion

Women who inject drugs are underrepresented in research—especially perinatal research—making it imperative that we actively seek out their perspectives. While we cannot determine which strategies were most effective and implementing all recommendations may not be possible for every study, these strategies were relatively easy to implement and likely created a trustworthy environment for participants to enrol, participate and recommend the study to their peers—small changes in pursuit of larger impact.

## Author Contributions

Each author certifies that their contribution to this work meets the standards of the International Committee of Medical Journal Editors. All authors made substantial contributions to the conception of this commentary and drafted and revised the work critically for important intellectual content. All authors gave final approval of the version to be published and agree to be accountable for all aspects of the work.

## Funding

This research is funded by a Health Systems Research Theme 2024 Collaborative Grant (UNSW Sydney) and the National Health and Medical Research Council (A.D.M., NHMRC EL1 Investigator Grant 2026888). G.K.B.‐M. is supported by the Commonwealth through an Australian Government Research Training Program Scholarship (DOI: https://doi.org/10.82133/C42F‐K220).

## Conflicts of Interest

Alison D. Marshall is a CI on a Gilead grant. Danielle M. Russell has received speaker fees from Gilead. The other authors declare no conflicts of interest.

## Data Availability

Data sharing is not applicable to this article as no datasets were generated or analysed during the current study.
